# CBFA2T2 is associated with a cancer stem cell state in renal cell carcinoma

**DOI:** 10.1186/s12935-017-0473-z

**Published:** 2017-11-14

**Authors:** Du-Chu Chen, You-De Liang, Liang Peng, Yi-Ze Wang, Chun-Zhi Ai, Xin-Xing Zhu, Ya-Wei Yan, Yasmeen Saeed, Bin Yu, Jingying Huang, Yuxin Gao, Jiaqi Liu, Yi-Zhou Jiang, Min Liu, Demeng Chen

**Affiliations:** 1 State Key Laboratory of Stress Cell Biology, School of Life Sciences, Xiamen University, Xiamen, 361005 Fujian China; 2 Department of Stomatology, Nanshan Affiliated Hospital of Guangdong Medical College, Shenzhen, 518060 Guangdong China; 3 Department of Oncology, Chinese PLA General Hospital, Beijing, 100853 China; 4 Institute for Advanced Study, Shenzhen University, Shenzhen, 518060 Guangdong China; 5 The First Affiliated Hospital of Sun Yat-Sen University, Guangzhou, 510275 Guangdong People’s Republic of China

**Keywords:** CBFA2T2, Renal cell carcinoma, Cancer stem cells, OCT-4, NANOG

## Abstract

**Background:**

Renal cell carcinoma (RCC) is the most common kidney cancer, accounting for approximately 80–90% of all primary kidney cancer. Treatment for patients with advanced RCC remains unsatisfactory. Rare cancer stem cells (CSCs) are proposed to be responsible for failure of current treatment.

**Methods:**

OncoLnc was used as a tool for interactively exploring survival correlations. Gene manipulation and expression analysis were carried out using siRNA, RT-PCR and Western blotting. Wound healing and invasion assays were used for phenotypical characterization. Aldefluor assay and FACS sorting Sphere culture were used to determine the “stemness” of CSCs. Co-Immunoprecipitation (Co-IP) was used to examine the interaction between OCT4 and CBFA2T2. Student’s t-test and Chi square test was used to analyze statistical significance.

**Results:**

CBFA2T2 expression can significantly predict the survival of RCC patients. Knocking-down of CBFA2T2 can inhibit cell migration and invasion in RCC cells in vitro, and reduce ALDH^high^ CSCs populations. CBFA2T2 expression is necessary for sphere-forming ability and cancer stem cells marker expression in RCC cell lines.

**Conclusions:**

Our data suggest that CBFA2T2 expression correlates with aggressive characteristics of RCC and CBFA2T2 is required for maintenance of “stemness” through regulation of stem cells factors, thereby highlighting CBFA2T2 as a potential therapeutic target for RCC treatment.

**Electronic supplementary material:**

The online version of this article (10.1186/s12935-017-0473-z) contains supplementary material, which is available to authorized users.

## Background

There are approximately 62,700 new cases of kidney cancer and an estimated 14,240 cancer associated deaths in the United States annually [1]. Renal cell carcinoma (RCC) represents one of the most common type of kidney cancer, accounting for 90% of adult renal malignancies [1]. Over the past five decades, the incidence of RCC has increased at a rate of 2% per year [2]. Surgery still serve as the standard way for localized and locally advanced RCC. Despite the surgical resection, the prognosis of advanced RCC is very poor, with a 5-year survival rate of 5–10% [3]. In addition, around 20–40% of RCC patients developed recurrence after treatment [4]. The mechanisms of how these metastasis and recurrence occur are largely uncharacterized. Recent studies have shown the presence of cancer stem cells (CSCs) in various solid tumor tissues [5–7]. CSCs are undifferentiated multipotential tumor cells that have high tumorigenic activity and the capacity to self-renew [5, 6, 8]. Moreover, CSCs promote cancer resistance to treatment and recurrence, leading to high mortality [6]. Therefore, CSC-targeted therapy can be an essential part of cancer therapy.

Previously, CSCs in RCC have been identified by using either stem cell markers [CD133, CD44, CXCR4, CD105 and Spalt-Like Transcription Factor 4 (SALL4)] [9–13] or functional assays (sphere-forming ability, side population and ALDH activity) [14–16]. Especially, in RCC cell lines, ALDH^high^ cells showed CSC properties in vitro, such as clonogenic and self-renewal ability and increased expression of OCT4 and NANOG [17]. Importantly, in the metastatic RCC cell lines, ALDH^high^ cells formed about 15% of the total number of cells and had higher sphere-forming capacity, self-renewal ability, and tumorigenicity than ALDH^low^ cells. While anther study showed that ALDH gene expression was correlated with tumor grade but not with tumor stage in patients with RCC [18]. Overall pooled analysis suggested that high CSCs markers, including CD133, CD44, CXCR4 and CD105, expression predicted poor overall survival, cancer-specific survival, disease-free survival and progression-free survival [19]. However, the molecular mechanism of how CSCs in RCC are maintained has not been fully explored.

Recently, corepressor CBFA2T2 has been shown to be involved in Prdm14-mediated germ cell formation through oligomerization to form a scaffold, which allows OCT4 to be stabilized on chromatin [20], indicating a role of CBFA2T2 in stem cell pluripotency regulation. CBFA2T2 has been known as a fusion partner of RUNX1 in acute myeloid leukemia [21], suggesting a role of CBFA2T2 in cancer development. Indeed, CBFA2T2 plays a key role in tumorigenesis in AOM/DSS colitis-associated carcinoma in mouse [22]. However, the role of CBFA2T2 in CSCs regulation remain unknown.

In this study, we sought to identify the role of CBFA2T2 in human RCC and to determine its characteristics in RCC tumorigenesis and RCC CSCs maintenance. Here, we showed that the survival rate in the RCC patients is negatively correlated CBFA2T2 expression. In addition, we showed the CBFA2T2 silencing impaired wound-healing and invasion capabilities of 786-O and A-498 cells, two human RCC cell lines. Our ALDH staining demonstrated that CBFA2T2 depletion in RCC cell lines led to reduced CSCs proportions. Importantly, CBFA2T2 is required for sphere-forming ability and cancer stem cells marker expression in RCC cell lines. We also showed an interaction between OCT4 and CBFA2T2 by pulldown assay. Collectively, our findings may provide new insights for future CSC study and clinical anti-cancer therapy in RCC.

## Results

### Association between CBFA2T2 and stem cell factors expression with the prognosis of RCC patients

**Fig. 1:**
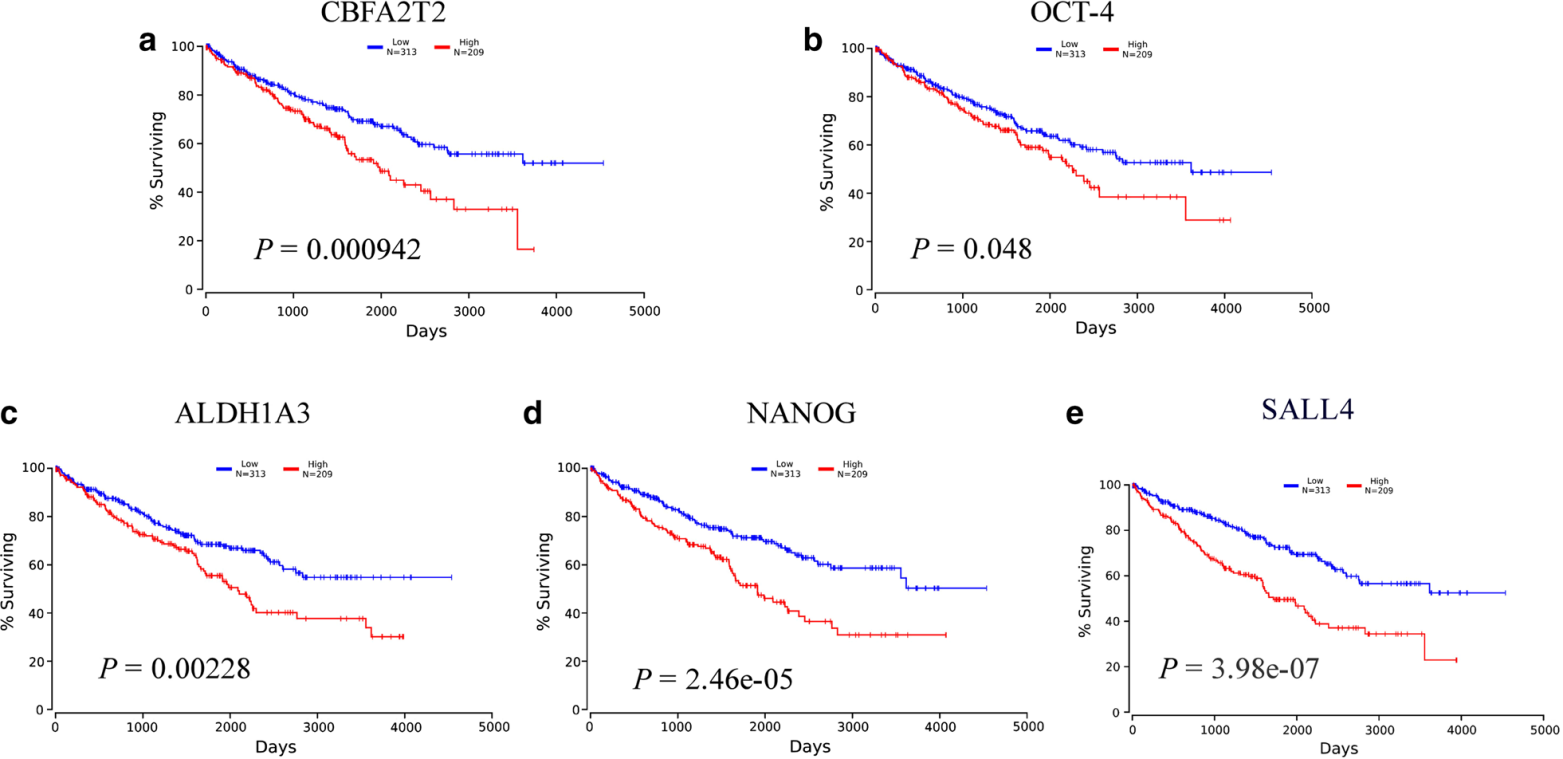
Kaplan-Meier survival curves of CBFA2T2 and stem cells marker expression in RCC. **a** RCC patients with high CBFA2T2 expression had shorter 5-year overall survival than those with low expression. **b** RCC patients with high OCT4 expression had shorter 5-year overall survival than those with low expression. **c** RCC patients with high ALDH1A3 expression had shorter 5-year overall survival than those with low expression. **d** RCC patients with high OCT4 expression had shorter 5-year overall survival than those with low expression. **e** RCC patients with high SALL4 expression had shorter 5-year overall survival than those with low expression

To examine the association of the expression levels of CBFA2T2, as well as several important cancer stem cells factors, including SOX2, OCT4, CD133, NANOG and ALDH1A3, with the and prognosis of patients with RCC, Kaplan–Meier survival analyses were performed on patients in the high and low expression groups between these gene expression and clinical outcomes on the homepage of oncolnc [23]. As shown in Fig. 1, the survival rate in the RCC patients who exhibited high CBFA2T2 expression was significantly reduced compared with patients with low CBFA2T2 expression by log-rank test (*p* = 0.000942). In the meantime, higher levels of OCT-4, ALDH1A3, NANOG and SALL4 expression were associated with worse survival (*p* = 0.048, *p* = 0.00228, *p* = 2.46e−05 and *p* = 3.98e−07, respectively) of RCC patients (Fig. 1b–e). However, the expression levels SOX2 and CD133 showed no significant correlation between survival rate of the RCC patients (data not shown). Indeed, our immunohistochemistry staining showed elevated expression of CBFA2T2 in RCC cancer compared to normal kidney tissue (Additional file 1: Figure S1). Our analysis using The Cancer Genome Atlas (TCGA) showed showing 0.4% of CBFA2T2—altered in RCC samples (Additional file 2: Figure S2). To investigate the relationship between CBFA2T2 expression and RCC further, we analyzed a cohort of 66 RCC patients. RCC were classified into 2 groups based on CBFA2T2 staining score: Low (0–1) and High [2, 3]. Our results indicated that increased expression of CBFA2T2 was associated with high grade RCC significantly (*p* = 0.023). Moreover, increased expression of CBFA2T2 was associated high stage disease, with a trend toward statistical significance (*p* = 0.104). However, our data showed that there were no significant differences for gender, lymph node, systematic metastasis and pathological types between the groups (Table 1).

**Table 1:** Relationship between clinicopathological features and CBFA2T2 expression

Variables	CBFA2T2 expression		*p* value
	Low (n = 31)	High (35)	
Gender			
Male	22 (71.4%)	25 (70.3%)	0.214
Female	9 (28.6%)	10 (29.7%)	
Fuhrman grade			
G1–2	14 (66.7%)	23 (65.7%)	0.023
G3–4	9 (33.3%)	3 (8.6%)	
Unknown	8 (23.5%)	9 (25.7%)	
pT stage			
pT1–2	16 (51.6%)	24 (68.6%)	0.104
pT3–4	8 (25.8%)	5 (14.3%)	
Unknown	7 (22.6%)	6 (17.1%)	
Lymph node metastasis			
Absence	26 (83.9%)	27 (77.1%)	0.382
Presence	2 (6.5%)	1 (2.9%)	
Unknown	3 (9.7%)	7 (20%)	
Systematic metastasis			
Absence	27 (87.1%)	27 (77.1%)	0.293
Presence	3 (9.7%)	4 (11.4%)	
Unknown	1 (3.2%)	4 (11.4%)	
Pathological type			
ccRCC	25 (80.6%)	25 (71.4%)	0.158
non-ccRCC	6 (19.4%)	10 (28.6%)	

### CBFA2T2 is required for migration and invasion of RCC cells in vitro

**Fig. 2:**
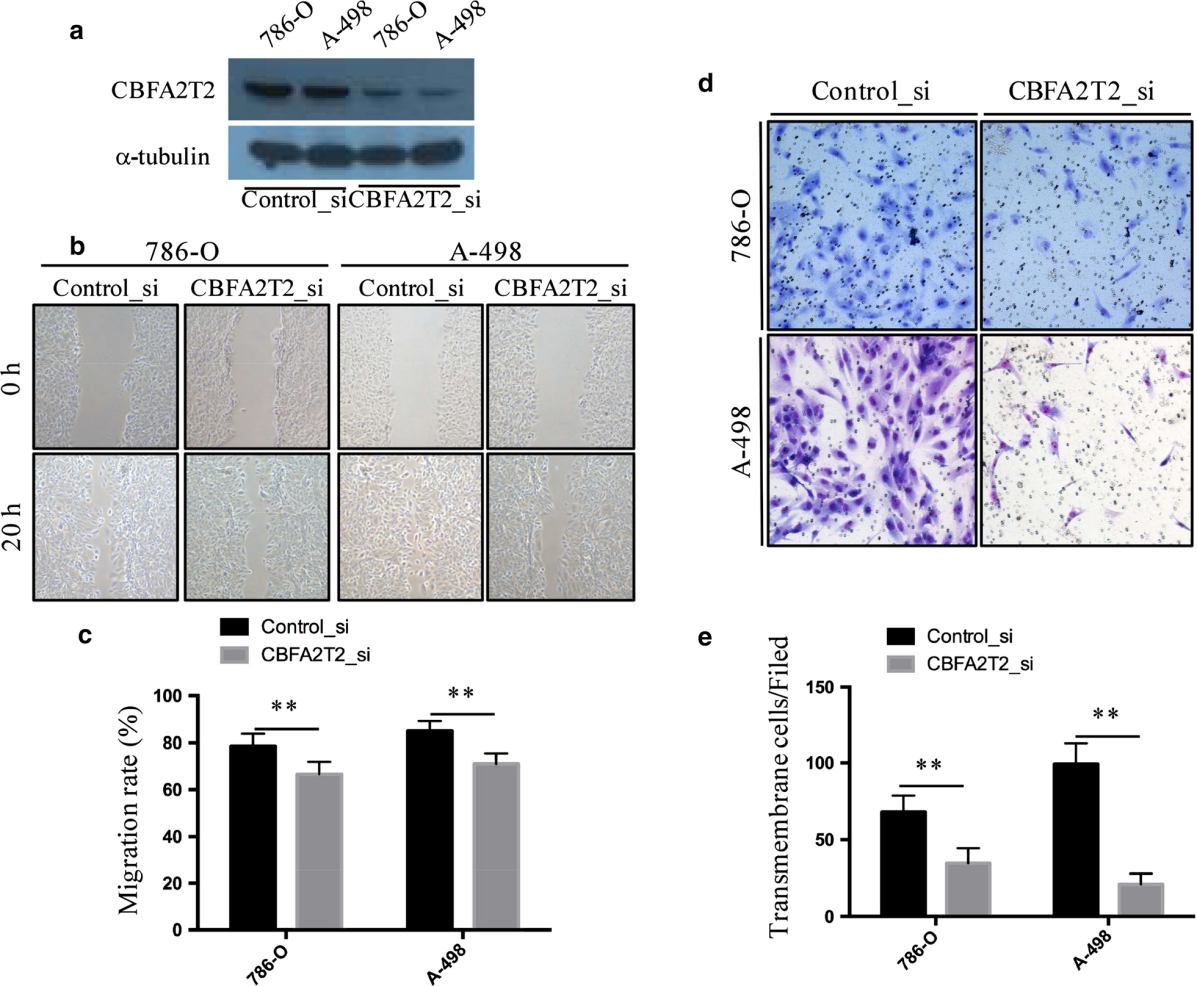
CBFA2T2 is required for migration and invasion activity of RCC cells. **a** Immunoblot assay of CBFA2T2 and β-actin proteins in 786-O and A-498 cells treated with siRNA against CBFA2T2 or control siRNA for 36 h. **b**–**c** 786-O and A-498 treated with siRNA against CBFA2T2 fill the wound area more slowly than those treated with control siRNA at 20 h. The wound-healing assay was expressed as wound closure rate (20 h average healed width divided by 0 h wound width). **d**–**e** CBFA2T2 silencing significantly blocked cell invasion of 786-O and A-498 cells. Images displaying the bottom side of the filter inserts with cells that migrated through the filter pores. The data are presented as the mean ± standard deviation with three independent experiments. **indicates p < 0.01

To investigate whether CBFA2T2 play a role in RCC cancer development, we used siRNA to inhibit CBFA2T2 expression. Knockdown of CBFA2T2 in both 786-O and A-498 cell were confirmed by Western blot analysis (Fig. 2a). Our wound healing assay showed that in silencing of CBFA2T2 in RCC cell lines significantly impaired their migratory abilities (Fig. 2b, c). Control 786-O cells almost completely closed the wound (78.5%) in 20 h, whereas 786-O cells treated with CBFA2T2 siRNA 20 h closed 66.7% of the wound. Similarly, control A-498 cells after 20 h almost completely closed the wound (85.0%), whereas A-498 cells treated with CBFA2T2 siRNA closed only 71% of the wound (Fig. 2b, c). In the invasion assay, we found that CBFA2T2 siRNA groups demonstrated a 31.51 ± 10.86% decrease in cell migration in 786-O cells and a 65.13 ± 9.97% decrease in that of A-498 cells, compared with the controls. These results indicated that CBFA2T2 reduced the migration and invasion in Renal cancer 786-O and A-498 cells.

### CBFA2T2 is required for maintenance of cancer stem cell property of RCC

**Fig. 3:**
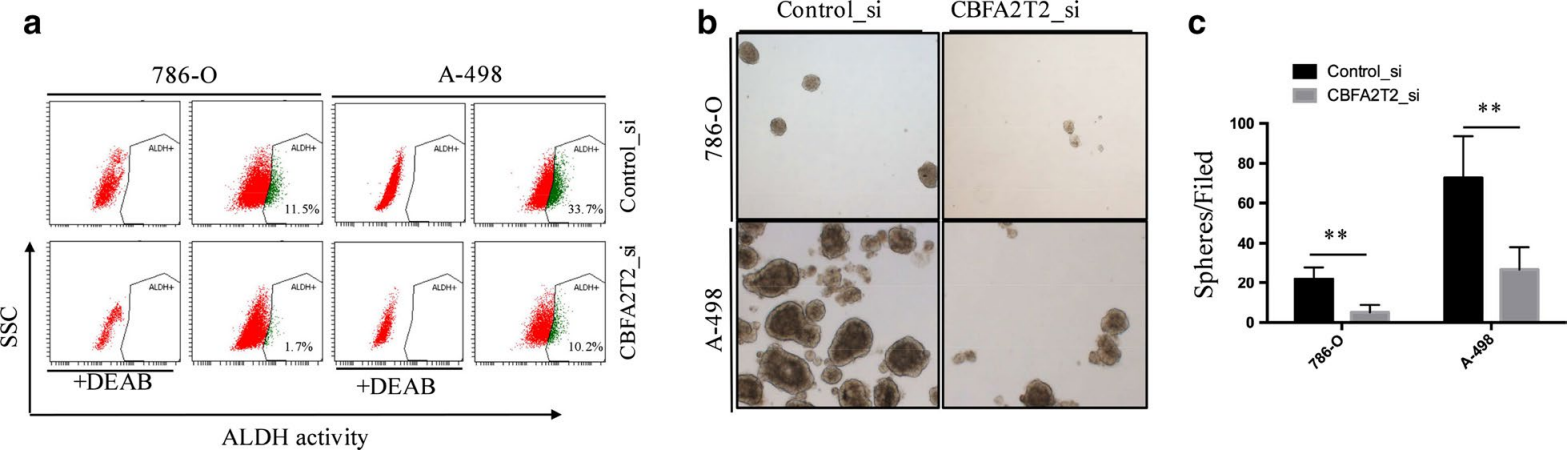
CBFA2T2 is required for cancer stem cell state of RCC cells. **a** 786-O and A-498 cells were treated with siRNA against CBFA2T2 or control siRNA for 24 h and subjected to the Aldefluor assay. Proportions of the ALDH-positive cell subpopulation in total cancer cells were indicated in the figure. **b**, **c** 786-O and A-498 cells were plated into ultra low-attachment 24-well plates at a density of 1000/well and cultured in tumor sphere medium containing siRNA against CBFA2T2 or control siRNA. Tumor spheres were photographed (**b**) and tumor sphere numbers were counted (**c**). The data are presented as the mean ± standard deviation with three independent experiments. **indicates p < 0.01

In RCC, ALDH^high^ subpopulation as assessed by the Aldefluor assay has been shown to be the cancer stem cells population, which has upregulated levels of OCT-4 and NANOG [17]. Hence, we examined whether knockdown of CBFA2T2 could lead to decrease of ALDH^high^ cells in vitro. As shown in our flow cytometry results (Fig. 3a), silencing of CBFA2T2 dramatically decreased the ALDH^high^ population of 786-O cells and A-498 cells. Next, we evaluated the effect CBFA2T2 on sphere-forming ability of sorted ALDH^high^ 786-O and A-498 cells in low-attachment plates. Depletion of CBFA2T2 decreased the number of spheres in sorted ALDH^high^ 786-O cells relative to the control group (21.92 ± 6.02 vs 5.17 ± 3.59, p < 0.01), while depletion of CBFA2T2 decreased the number of spheres in sorted ALDH^high^ A-498 cells relative to the control group (72.67 ± 20.95 vs 26.92 ± 11.24, p < 0.01) (Fig. 3b, c). This further supports that CBFA2T2 is required for maintenance of cancer stem cell property in RCC.

### CBFA2T2 is required for ALDH1A3, OCT-4 and NANOG expression in CSCs of RCC

**Fig. 4:**
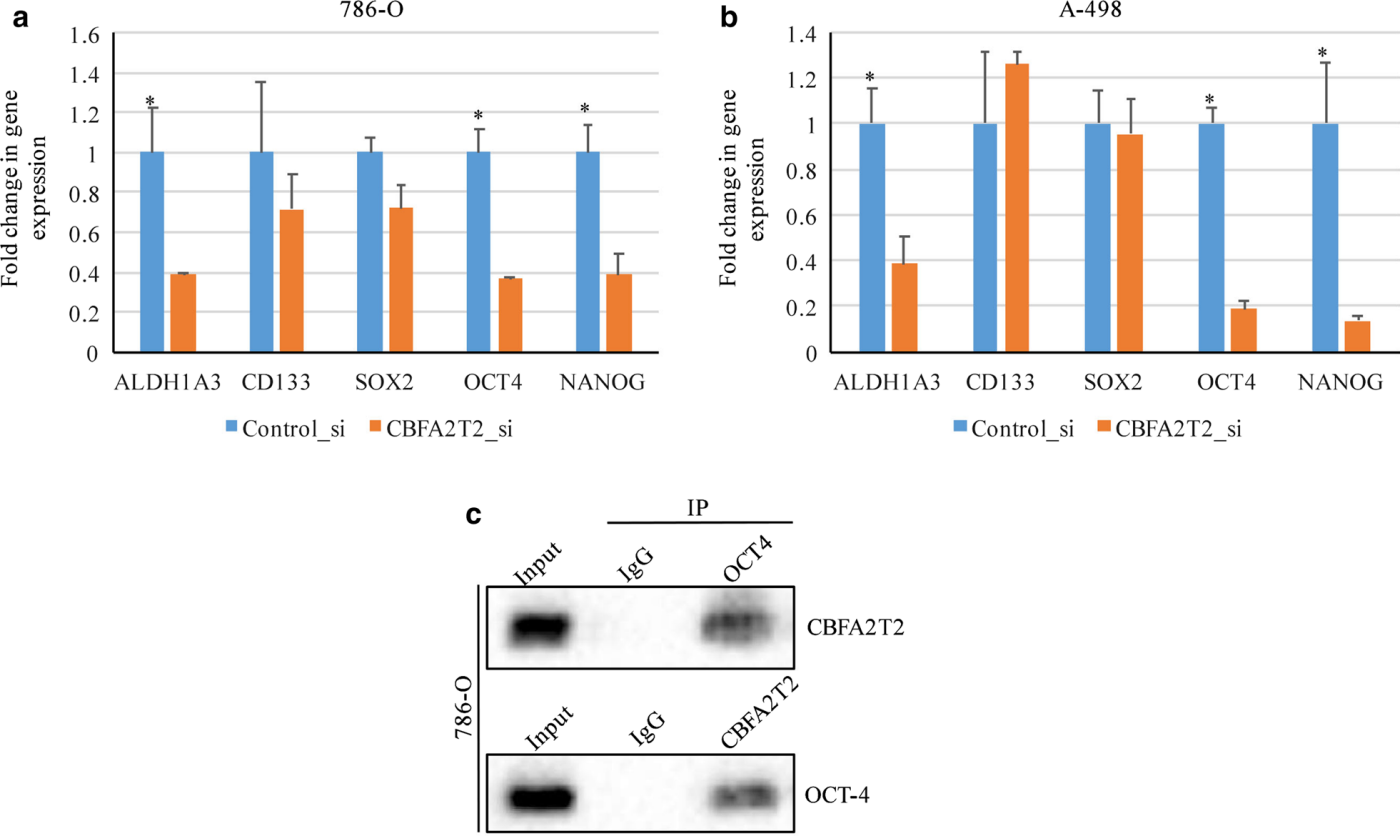
CBFA2T2 is required for the expression of known cancer stem cell markers in RCC cells. **a** Quantitative real-time RT-PCR analysis for the expression of ALDH1A3, CD133, OCT4, Sox2, and Nanog mRNAs in 786-O cells after siRNA treatment for 48 h. P < 0.05 was considered as significant. **b** Quantitative real-time RT-PCR analysis for the expression of ALDH1A3, CD133, OCT4, Sox2, and Nanog mRNAs in A-498 cells after siRNA treatment for 48 h. p < 0.05 was considered as significant. **c** The interaction between OCT4 and CBFA2T2 in CSCs population showing by pull-down assay

Previous reports have indicated that ALDH^high^ cells showed elevated expression of OCT-4 and NANOG. To investigate whether CBFA2T2 was involved in stem cell marker genes expression of RCC CSCs, we first isolated ALDH^high^ 786-O and A-498 cells. We maintained these cells as sphere in stem cells medium and treated with siRNA. We then examined the levels of these markers using quantitative RT-PCR approach. The qRT-PCR results confirmed that depletion of CBFA2T2 downregulated ALDH1A3, OCT-4 and NANOG expression in CSCs of 786-O and A-498 cells (Fig. 4a, b). Although, we didn’t detect any expression difference between two known CSC markers: CD133 and SOX2 (Fig. 4a, b). These findings suggest that CBFA2T2 maintained CSCs property by regulation of ALDH1A3, OCT4 and NANOG in RCC cell lines. Since CBFA2T2 normally functions as a transcription regulator, we reasoned that it may act as cofactor of stem cell transcriptional factor. To investigate the mechanism on how CBFA2T2 regulates CSCs in RCC, we examined the interaction between CBFA2T2 and OCT4. We found that CBFA2T2 was able to be pulled down by immunoprecipitation with OCT4 (Fig. 4c), suggesting it might serve as cofactor of OCT4 in regulating the stemness of CSCs population in RCC.

## Discussion

Cancer stem cells research is expanding rapidly based on the knowledge of CSCs from various cancer types. Understanding of how CSCs are regulated holds great implication in therapeutic resistance and relapse after initial therapy. In current study, we demonstrated that CBFA2T2 is an indication of the survival of RCC patients. In addition, our functional data supported a role of CBFA2T2 in ALDH^high^ CSCs population isolated from RCC cell lines. Also, we showed that CBFA2T2 is required for expression of stem cells factors in ALDH^high^ CSCs population.

In this report, we demonstrate that CBFA2T2 is required for CSCs maintenance in RCC cell lines. CBFA2T2 has been shown as an important factor for efficient inflammatory-associated colonic carcinogenesis [22]. In acute myeloid leukemia, CBFA2T2 has been identified as a fusion partner of RUNX1 [21]. Interestingly, fusion RUNX1/CBFA2T2 transcript is highly upregulated compared with wild-type CBFA2T2 gene [21], suggesting that CBFA2T2 alone might not be sufficient for the development of acute myeloid leukemia.

In a metastatic RCC cell line, the expression of the OCT-4 and NANOG gene was significantly overexpressed compared to all other RCC cell lines [24]. Our analysis also showed that higher levels of OCT-4 and NANOG expression were associated with worse survival of RCC patients, indicating these stem cell pluripotency factors might be a common pathway for inhibition of CSCs.

Our data that CBFA2T2 could be pull down by immunoprecipitation with OCT4, suggesting it might serve as cofactor of OCT4 in regulating the stemness of CSCs population in RCC. Since OCT4 can regulate the expression of NANOG [25], it is highly possible that the interaction of OCT4 and CBFA2T2 is required for NANOG expression.

In summary, our results established a link between expression of CBFA2T2 and clinical prognosis in RCC. Our data also showed that the CBFA2T2 plays a critical role in the maintenance of CSC properties in human RCC cells.

## Conclusion

We showed in our study that CBFA2T2 is required for maintenance of “stemness” through regulation of stem cells factors and CBFA2T2 could be a potential therapeutic target for RCC treatment.

## Materials and methods

### Cell culture and siRNA silencing

786-O and A-498 cell lines (ATCC, USA) were cultured in DMEM supplemented with 10% fetal bovine serum, penicillin (5000 U/ml) and streptomycin (5000 μg/ml) (Thermo Fisher Scientific, China) at 37 °C with 5% CO_2_. For siRNA assay, pool siRNAs consist of three to five target-specific 19- to 25-nucleotide siRNAs designed to knock down CBFA2T2 gene expression or scrambled siRNA were purchased from Santa Cruz Biotechnology. Monolayer or sphere 786-O and A-498 cells were transfected into cell line using Lipofectamine™ 2000 (Invitrogen) according to the manufacturer’s instructions.

### Wound healing and invasion assays

For wound healing assay, 786-O and A498 cells transfected with the siRNAs against CBFA2T2 or control siRNA were plated on 6-well plates. When cells become confluent, cells were scraped with a plastic pipette tip [26]. The wound closure was photographed at different time points (0 and 20 h after scraping). For invasion assay, 786-O and A-498 cells were transfected with the siRNAs for 48 h, and then digested and resuspended at 10^5^ cells/ml. 200 μl of cell suspension was transferred into insert of the Transwell covered with Matrigel (BD Bioscience). After 48 h, the cells that migrated through the permeable membrane were fixed with paraformaldehyde, stained with haematoxylin and eosin, and finally counted. Aphidicolin (Sigma) was used to inhibit proliferation of cells during wound healing and invasion assay. Experiments were performed in triplicate.

### Aldefluor assay and FACS sorting

The ALDH activity of 786-O and A-498 cells was determined by using the Aldefluor assay kit (Stem Cell Technologies) following the manufacturer’s guidance. Briefly, cells were dissociated into single cells by trypsin/EDTA digestion, washed twice in PBS and suspended in 1 ml Aldefluor assay buffer containing 5 μl ALDH substrate and incubated for 30 min at 37 °C in the dark. Diethylaminobenzaldehyde (DEAB) was added to a separate aliquot of the sample as negative control. Cells were then washed twice with buffer and maintained in ALDH buffer on ice. For FACS sorting, cells were sorted using an Aria II cell sorter (BD Biosciences). The sorting gates were established, by negative control cells which were treated with the ALDH inhibitor DEAB. Cells were sorted into DMEM medium containing 20% FBS, penicillin (5000 U/ml) and streptomycin (5000 μg/ml).

### Sphere culture

After FACS sorting, cells were then inoculated into 1:1 DMEM/F12 medium containing N2 supplement (100x, invitrogen), B27 supplement (50x, invitrogen), human recombinant epidermal growth factor (EGF; 10 ng/ml) and basic fibroblast growth factor (bFGF; 10 ng/ml) without serum at a density of 10,000 cells/well in ultra-low-attachment six-well plates (Corning, Inc., Corning, NY, USA). Fresh aliquots of stem cell medium were added every other day.

### Real-time PCR and Western blot analysis

All sphere samples after siRNA treatment were centrifuged for 10 min at 300×*g* and the supernatant was removed. Total RNA was extracted with Trizol (Life Technologies, China) and reverse-transcribed with a first-strand cDNA synthesis kit (Roche Diagnostics). TaqMan PCR was performed as described [27, 28]. Stem cell marker mRNA levels were standardized by GAPDH levels and expressed as relative ratio to those of sphere cells treated with control siRNA. Primer sequences are:

ALDH1A3 forward: 5′-TGAATGGCACGAATCCAAGAG-3′

ALDH1A3 reverse: 5′-CACGTCGGGCTTATCTCCT-3′

PROM1 forward: 5′-AGTCGGAAACTGGCAGATAGC-3′

PROM1 reverse: 5′-GGTAGTGTTGTACTGGGCCAAT-3′

SOX2 forward: 5′-GCCGAGTGGAAACTTTTGTCG-3′

SOX2 reverse: 5′-GGCAGCGTGTACTTATCCTTCT-3′

OCT4 forward: 5′-CTGGGTTGATCCTCGGACCT-3′

OCT4 reverse: 5′-CCATCGGAGTTGCTCTCCA-3′

NANOG forward: 5′-TTTGTGGGCCTGAAGAAAACT-3′

NANOG reverse: 5′-AGGGCTGTCCTGAATAAGCAG-3′

GAPDH forward: 5′-GGAGCGAGATCCCTCCAAAAT-3′

GAPDH reverse: 5′-GGCTGTTGTCATACTTCTCATGG-3′

For Western blot assay, 786-O and A498 cells transfected with the siRNAs against CBFA2T2 or control siRNA for 72 h were washed two times with ice cold phosphate-buffered saline (PBS) and lysed in RIPA buffer (50 mM Tris pH 7.4, 250 mM NaCl, 5 mM EDTA, 1% NP-40, 0.1% SDS, 0.5% sodium deoxycholate, 1 mM phenylmethylsulphonyl fluoride) containing 1% protease inhibitor cocktail (Roche) [29]. Cell lysates were centrifuged at 12,000×*g* for 10 min at 4 °C. Supernatant were collected for protein concentration measure using the BCA protein assay kit (Pierce). Total protein of 15 μg was subjected to SDS-PAGE, transferred to polyvinylidene fluoride (PVDF) membrane, and incubated with antibodies, followed by HRP-conjugated secondary antibodies. Specific proteins were detected by ECL Western blotting Detection Reagents (GE Healthcare Biosciences). Antibody against CBFA2T2 was purchased from Abcam (ab128164); antibody against α-tubulin was the products of Sigma-Aldrich (clone B-5-1-2).

### Kaplan–Meier survival curves analysis

In this study, OncoLnc (http://www.oncolnc.org) was used as a tool for interactively exploring survival correlations [23]. OncoLnc dataset contains survival data for 522 patients from kidney renal clear cell carcinoma (KIRC) cancer studies performed by The Cancer Genome Atlas (TCGA). The multivariate cox regressions were performed followed by a Kaplan–Meier analysis for CBFA2T2, OCT-4, ALDH1A3 and NANOG.

### Statistical analysis

For statistical analysis, GraphPad Prism (version 7) was used. Student’s t-test was used to analyze statistical significance of the data. For Kaplan–Meier Survival, p-value represents the results of log-rank test. Chi square test was used for analyzing the correlation between clinicopathologic categories and CBFA2T2 expression. A p-value of less than 0.05 was considered to be statistically significant.

## Additional files

**Additional file 1: Figure S1.** CBFA2T2 expression is elevated in RCC tissues. (A) Representative immunostaining of CBFA2T2 in normal kidney tissue. (B) Representative immunostaining of CBFA2T2 in ccRCC. (C) CBFA2T2 protein expression in RCC samples was significantly higher than that of normal kidney tissues. **p  < 0.01.

**Additional file 2: Figure S2.** The Cancer Genome Atlas (TCGA) analysis. (A) Analysis of TCGA data set showing 0.4% of CBFA2T2—altered in RCC samples.
