# Correction to: CBFA2T2 is associated with a cancer stem cell state in renal cell carcinoma

**DOI:** 10.1186/s12935-018-0564-5

**Published:** 2018-05-11

**Authors:** Du-Chu Chen, You-De Liang, Liang Peng, Yi-Ze Wang, Chun-Zhi Ai, Xin-Xing Zhu, Ya-Wei Yan, Yasmeen Saeed, Bin Yu, Jingying Huang, Yuxin Gao, Jiaqi Liu, Yi-Zhou Jiang, Min Liu, Demeng Chen

**Affiliations:** 1 State Key Laboratory of Stress Cell Biology, School of Life Sciences, Xiamen University, Xiamen, 361005 Fujian China; 2 Department of Stomatology, Nanshan Affiliated Hospital of Guangdong Medical College, Shenzhen, 518060 Guangdong China; 3 Department of Oncology, Chinese PLA General Hospital, Beijing, 100853 China; 4 Institute for Advanced Study, Shenzhen University, Shenzhen, 518060 Guangdong China; 5 The First Affiliated Hospital of Sun Yat-Sen University, Guangzhou, 510275 Guangdong People’s Republic of China

## Correction to: Cancer Cell Int (2017) 17:103 10.1186/s12935-017-0473-z

Upon publication of the original article [1] it was highlighted by the authors that a grant awarded to support the research work of the study was missed in the Funding Acknowledgements of their manuscript. It should also be acknowledged that the grant titled “*Shenzhen healthcare research project” (201505025 to Youde Liang)* also contributed to the resources for this research. This has since been formally noted in this correction article.
