# Supplementary figures and images for: CBFA2T2 is associated with a cancer stem cell state in renal cell carcinoma
Source: Cancer Cell Int. 2017 Nov 14;17:103. doi: 10.1186/s12935-017-0473-z (PMC5686942; doi:10.1186/s12935-017-0473-z)

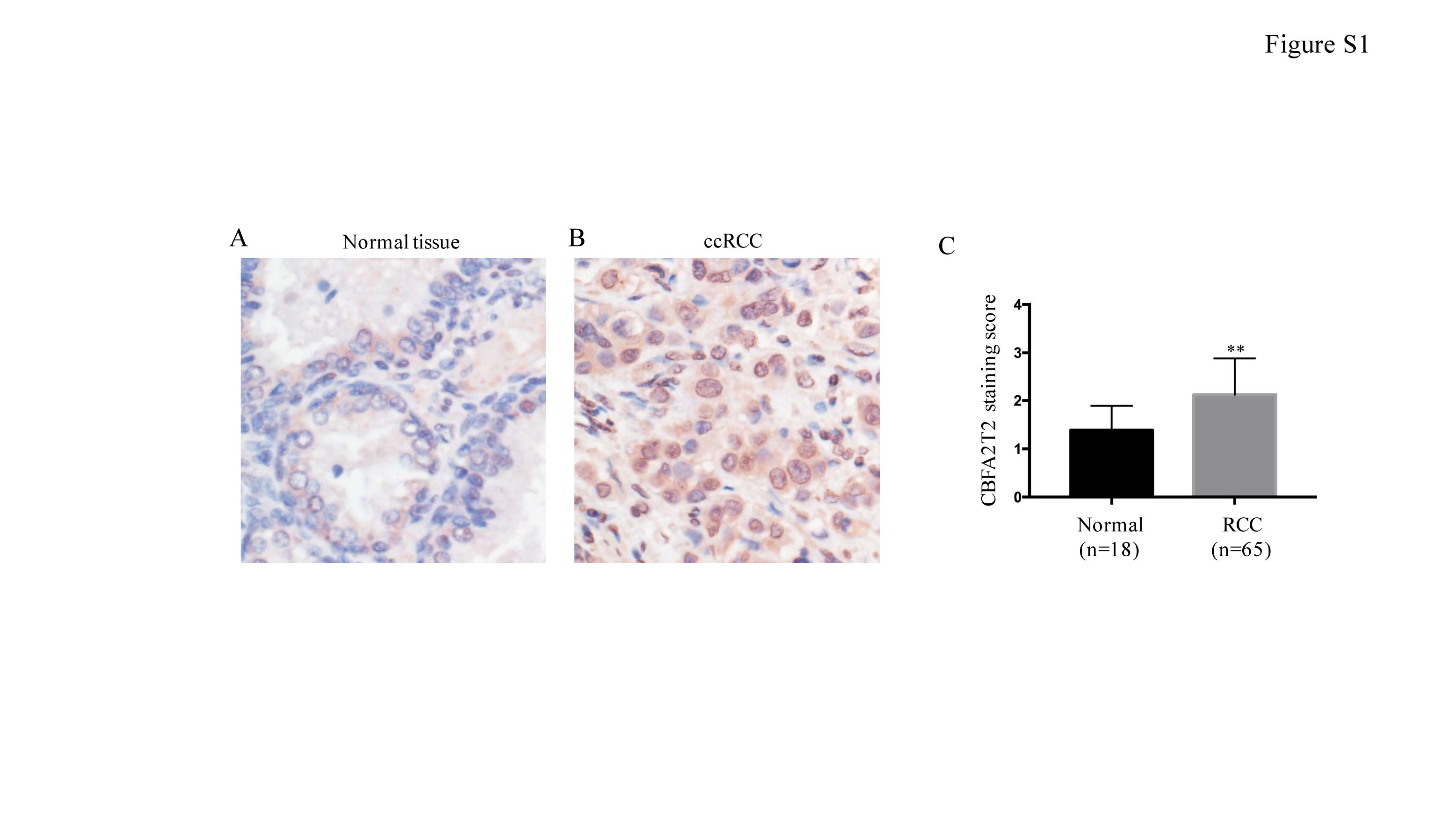

Supplement: Supplementary file 1 — Additional file 1: Figure S1. CBFA2T2 expression is elevated in RCC tissues. (A) Representative immunostaining of CBFA2T2 in normal kidney tissue. (B) Representative immunostaining of CBFA2T2 in ccRCC. (C) CBFA2T2 protein expression in RCC samples was significantly higher than that of normal kidney tissues. **p < 0.01. [file 12935_2017_473_MOESM1_ESM.jpg]

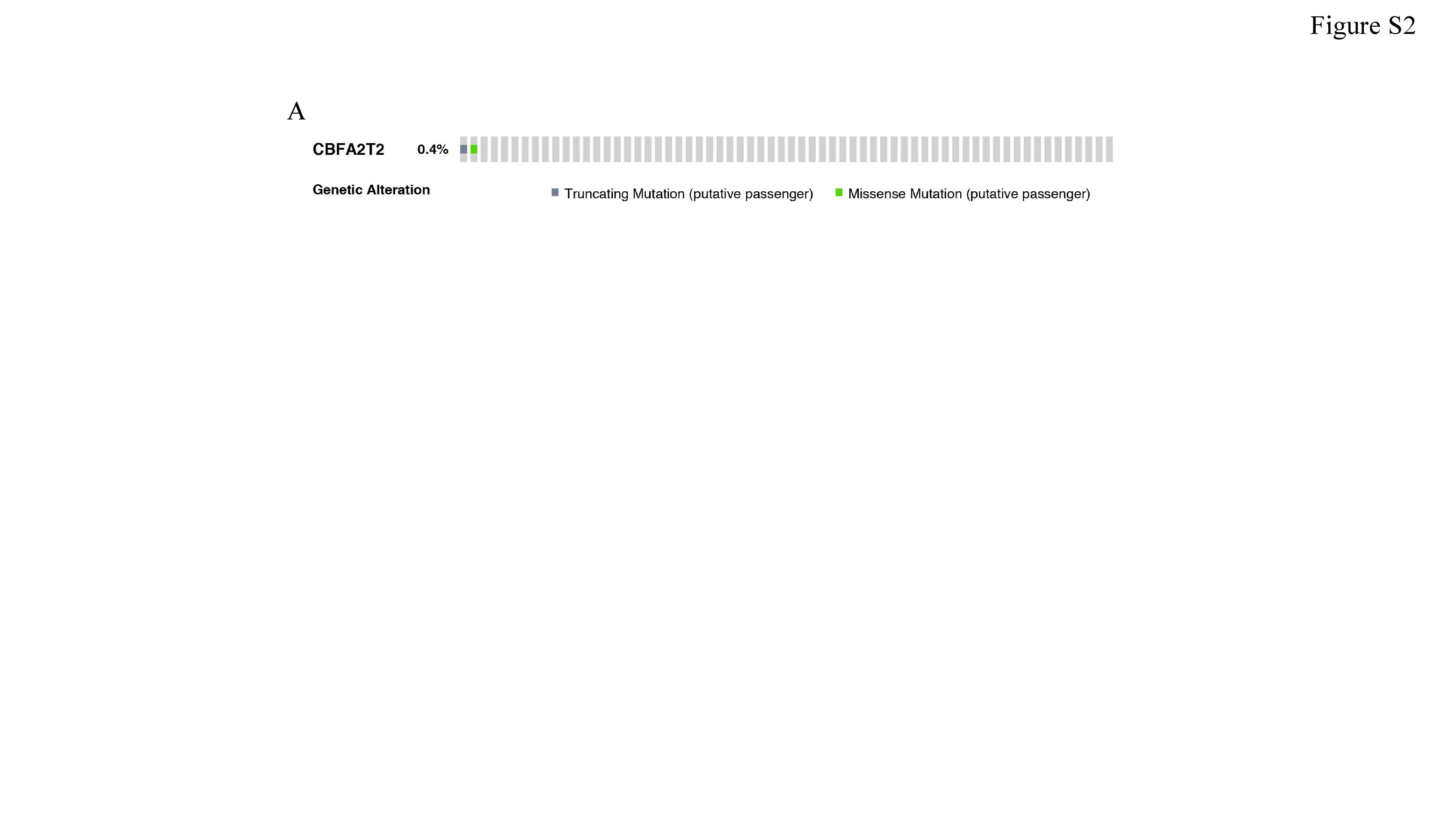

Supplement: Supplementary file 2 — Additional file 2: Figure S2. The Cancer Genome Atlas (TCGA) analysis. (A) Analysis of TCGA data set showing 0.4% of CBFA2T2—altered in RCC samples. [file 12935_2017_473_MOESM2_ESM.jpg]
